# ADHD Patients with Suicidal Behaviour: Risk Factors, Comorbidities, and Clinical Profile: A Naturalistic Exploratory Study

**DOI:** 10.3390/brainsci14050437

**Published:** 2024-04-27

**Authors:** Bryan Diaz-Piedra, Joseph Sadek

**Affiliations:** 1 Faculty of Science, Dalhousie University, Halifax, NS B3H 4R2, Canada; bryandiazpiedra@gmail.com; 2 Department of Psychiatry, Faculty of Medicine, Dalhousie University, Halifax, NS B3H 4R2, Canada

**Keywords:** attention deficit hyperactivity disorder, ADHD, suicide, suicidality, suicide ideation, psychiatric disorders, prevention, risk factor

## Abstract

Attention deficit hyperactivity disorder (ADHD) is a common neurodevelopmental disorder that disrupts brain functioning and is characterized by hyperactivity, impulsivity, and inattention. With varying presentations and complex etiological factors contributing to the development of ADHD, along with its persistence into adulthood, ADHD must be studied. Researchers have taken an interest in the relationship between ADHD and suicide, which is a serious public health concern with increasing prevalence rates in the Americas. The current literature reveals conflicting views on the importance of psychiatric comorbidities in the development of suicidal behaviours in ADHD patients. Therefore, this study aimed to determine whether there were significant differences between adult ADHD patients with suicide risk and adult ADHD patients without suicide risk. This study was a naturalistic retrospective chart review pilot study that used a sample of adults with a confirmed diagnosis of ADHD from January 2023 to August 2023. Using convenience sampling and sets of inclusion and exclusion criteria, patient data were sequentially collected from Med Access electronic medical records. The control and experimental groups each consisted of 50 patients (100) ranging from 19 to 58 years old. Our quantitative data were analyzed using non-parametric statistical tests, including the Chi-Square test and the Mann–Whitney U test. The results showed significant associations between ADHD patients with suicide risk and (1) borderline personality disorder; (2) binge eating disorder; (3) seven specific psychosocial risk factors; and (4) a higher number of antidepressant medication trials. No significant associations were found with other psychiatric disorders; however, there are important sex differences in terms of the risk factors. Our pilot study reveals several significant differences between adult ADHD patients with suicide risk and those without suicide risk. However, given our limited sample size and limitations, we hope our study encourages larger-scale studies to further investigate this relationship to improve its generalizability.

## 1. Introduction

### 1.1. Attention Deficit Hyperactivity Disorder

Attention deficit hyperactivity disorder (ADHD) is a neurodevelopmental disorder that generally leads to disruptions in brain functioning and is characterized by hyperactivity, impulsivity, and inattention [1]. ADHD is one of the most common psychiatric disorders in children and adolescents [2], with a global prevalence of 8% in children and adolescents [3] and 3.4% in adults [4]. Its prevalence in adults is significantly lower than in children and adolescents because only about 50% of cases progress into adulthood [5]. In the general population, men are more commonly diagnosed with ADHD compared to women; however, the difference is likely overestimated given that women are believed to be underdiagnosed [1]. Cultural differences also exist in ADHD prevalence because of varying diagnostic and methodological practices, in addition to varying attitudes regarding children’s behaviour [6]. For example, in the United States, clinical identification of ADHD in African American and Latino populations is typically lower than in Caucasian populations, indicating the need for culturally sensitive ADHD diagnostic methods [6].

ADHD can be classified into three main presentations according to the Diagnostic and Statistical Manual of Mental Disorders, Fifth Edition (DSM-5), which include the combined presentation, the predominantly inattentive presentation, and the predominantly hyperactive–impulsive presentation [6]. The prevalence of each type varies, with roughly 62% of adults having the combined presentation, 31% having the inattentive presentation, and only 7% having the hyperactive–impulsive presentation [7]. In previous editions of the DSM, ADHD was divided into three subtypes; however, this shift in ADHD presentations suggests that the symptoms people experience are fluid, meaning they can change across their lifetime [8]. As outlined in the DSM-5, to be diagnosed with ADHD, a minimum of six DSM-specified symptoms in children and young adolescents and five DSM-specified symptoms in older adolescents and adults (aged ≥ 17) must be present for a minimum of six months. The difference in the diagnostic threshold for older adolescents and adults is to ensure that ADHD is effectively diagnosed in these age groups [6]. Additionally, these symptoms must have been present before the age of 12 and in two or more settings (e.g., at work and school), while negatively impacting the quality of occupational, social, and academic functioning [6].

Inattentive symptoms include forgetfulness and high distractibility, in addition to difficulty focusing, paying attention, organizing activities, following instructions, and listening when spoken to [6]. Hyperactivity refers to inappropriate and excessive motor movements that are disruptive to everyday functioning and include fidgeting, the inability to remain seated, running, climbing, and talking [1]. Lastly, impulsive symptoms consist of performing activities with the potential for harm without thinking about their consequences [6,9]. Impulsive behaviours also reflect the desire for immediate gratification or rewards and can also manifest as social intrusiveness depending on the activities performed. Hyperactivity becomes less prominent as children and adolescents age, but inattentive and impulsive symptoms typically persist into adulthood [6]. Important to this study are the impulsive symptoms, as the varying degrees of impulsivity in adults with ADHD have generated concern in researchers about the relationship between impulsivity in ADHD and suicidal behaviours [9,10].

The etiology of ADHD is complex and involves the interaction of multiple factors, including environmental and genetic factors, brain abnormalities [1], and physiological changes [6]. ADHD is highly heritable, with first-degree biological relatives being at a considerably greater risk of developing ADHD. There is not one specific gene that leads to the development of ADHD but rather a variety of susceptibility genes, such as irregularities in the dopamine D4 and D5 receptor genes, that may increase the risk of ADHD [1]. Moreover, research has shown that certain environmental exposures before birth (prenatal) and during birth (perinatal), such as low birth weight and preterm birth, can increase the risk of ADHD for up to 40 years after birth [11]. 

### 1.2. Suicide

Suicide is a serious public health concern, accounting for approximately 700,000 deaths every year [12]. Over the past 20 years, global suicide mortality rates have decreased [13]; however, rather than decreasing, the rates in the Americas have gradually increased [14]. These increasing rates suggest that additional focus should be directed towards preventing suicidal behaviours (SBs). SB encompasses several actions and exists on a spectrum from less severe forms of suicidal ideation (SI) to completed suicide [15]. Other behaviours that are included in SB are suicidal gestures and self-injurious behaviours [16], like attempted overdoses or cutting that causes visible wounds. SI can manifest as either passive or active forms. Passive SI involves thoughts of death and wishes to die without a plan or actual intentions of dying, while active SI involves thoughts and intentions of taking one’s life with an established method and plan [15]. SI differs from suicide attempts (SAs), which are likely self-injurious behaviours that are performed with the intention of dying and can result in a completed suicide [17]. 

The causes of SB are multifactorial and involve a variety of factors that typically increase the risk of completed suicides. Suicide’s risk factors can be either static or dynamic. Static risk factors are those that are fixed and do not change over the lifespan and include sex, race, and age. Dynamic risk factors are not static and can fluctuate to either increase or decrease the risk of suicide depending on access to healthcare, impulsivity, firearm possession, and substance use, among other factors [18]. In terms of sex, men are at a greater risk of dying by suicide than women, often because they utilize more lethal methods, such as firearms or hanging. Conversely, women are at a lower risk of suicide but are overall more likely to attempt suicide [18,19]. A few other important risk factors include previous psychiatric history, previous SAs, and being a member of the geriatric population [19]. For example, members of the geriatric population may experience elevated feelings of loneliness and isolation, which increases suicidal thoughts, leading to elevated suicidal risk [18]. In addition, studies examining suicide show that a previous SA significantly increases the risk of a fatal suicide, where 1 in 19 previous attempters complete suicide [19]. 

### 1.3. Rationale

Previous research has found that ADHD may be associated with suicide [10,16,20,21,22]. Of these studies, some highlight the importance of comorbid psychiatric disorders in the development of suicidal behaviours, with depression being a common risk factor [16,20,23]. However, other studies suggest that psychiatric comorbidities do not play a role in the development of suicidal behaviours in this population [10,24,25]. These contrasting views suggest that additional research is required to better understand the potential risk factors and the involvement of comorbid psychiatric disorders in the development of suicidal behaviours in adults with a diagnosis of ADHD. Additionally, there has also been growing interest in the association between self-harm, suicidal behaviours, and ADHD [9,17]. These studies recommend that these relationships should be further investigated to better understand whether there are any significant associations. This study has the potential to contribute to our understanding of self-harm, suicidal behaviour, and ADHD by improving our ability to identify risk factors and determine the significance of comorbid psychiatric disorders, which is a topic that is largely debated in the current literature. Further, studies have previously highlighted risk factors for suicide in ADHD patients; however, we hope to identify various risk factors that will further the clinical profile of these patients to improve clinicians’ ability to identify suicide risk in this patient cohort. This is important as suicide is still an ongoing problem, and identifying both risk factors and comorbid disorders can improve suicide detection, which is a major goal of our study. 

### 1.4. Objectives

The primary goal of this study was to determine whether there were significant differences in adult ADHD patients with suicide risk compared to adult ADHD patients without suicide risk. In addition, we aimed to further develop the clinical profiles of these patients so that those at risk of suicide can be better identified and protected with the use of suicide intervention practices. Our findings will contribute to the ongoing debate existing in the current literature regarding the association between comorbid disorders and suicide risk and will further outline important risk factors associated with suicide in ADHD patients. 

We hypothesized that ADHD patients with suicide risk would have more psychiatric comorbidities, medical problems, antidepressant medication trials, and poorer social abilities when compared to adult ADHD patients without suicide risk.

## 2. Methods

### 2.1. Study Design

This study was a naturalistic retrospective chart review exploratory study of adults with a confirmed diagnosis of ADHD with and without suicide risk from January 2023 to August 2023. During this time, patients were screened by a psychiatrist at an outpatient psychiatric clinic in Dartmouth, Nova Scotia. Patients were then selected for this study based on whether they met the inclusion or exclusion criteria. The naturalistic nature of this study consisted of completing the patient intake form in a real-life clinical environment without manipulating any variables. Those who attended the psychiatric clinic received their standard of care and were not questioned under experimental conditions. By using this approach, our goal was to reduce patient stress and gather genuine and accurate responses from patients. Moreover, given the retrospective study methodology and the 100-patient sample size we used for this study, it was not feasible for all the patients to return to the psychiatric clinic to complete and sign a consent form. A waiver of consent was submitted and approved by the Nova Scotia Health Research Ethics Board (REB). As we met all the requirements, the patients were not required to provide consent for their de-identified medical data and personal health information (PHI) to be used in our retrospective chart review. The REB provided ethics approval for this study (REB File #1029517). 

Our study used convenience sampling to gather 100 patients for inclusion in this study. Convenience sampling is a non-probability form of sampling that involves selecting clinical cases that are easily accessible by researchers [26]. This method of sampling was used as we only had access to the files of patients who had previously attended this clinic. This study is one of the few that has examined the relationship between ADHD and suicide risk in adults living in Nova Scotia, which is why we performed a smaller-scale pilot study first to look for any significant findings before performing a larger, more intensive study. Additionally, convenience sampling is commonly used in pilot studies and does not allow the results to be generalized to the general population [26]. Therefore, we hope that our study encourages larger-scale studies with greater representative sample sizes to ensure that the findings can be generalized to all ADHD patients living in Nova Scotia. 

### 2.2. Study Setting and Population

This study enrolled outpatient psychiatric clinic patients with a confirmed diagnosis of ADHD, with or without suicide risk. A total of 100 patients from the Dartmouth South Professional Centre were included in this study, with 50 patients in each study group. The experimental group had 50 adult ADHD patients with suicide risk, whereas the control had 50 adult ADHD patients without suicide risk. Considering the total number of ADHD patients with suicide risk screened at the outpatient psychiatric clinic, we decided to include 50 patients in each study group to ensure that both groups were full given the outlined inclusion and exclusion criteria below. Moreover, each study group consisted of 25 females and 25 males to allow for fair comparisons between the groups.

The initial patient intake took place at the Dartmouth South Professional Centre in Dartmouth, Nova Scotia. At this clinic, there are roughly 1500 adult ADHD patients screened each year, with approximately 300 of them presenting with suicide risk. The majority of the patients are from the Central geographic management zone, which includes the Halifax area, Eastern Shore, and West Hants. The patients who visit the clinic typically range from 25 to 40 years old, and females are more likely to visit the clinic compared to males. 

### 2.3. Inclusion and Exclusion Criteria

For patients to be included in this study, they were required to meet the inclusion criteria for either the experimental or control group. The inclusion criteria for the experimental group required patients to (1) have a confirmed diagnosis of ADHD made by a psychiatrist according to the DSM-5 criteria; (2) have a past or present history of self-harm or suicide attempts, suicidal ideation, or a suicidal plan as self-reported on the Dr. Sadek Psychiatric Assessment Intake Form; and (3) be males and females over the age of 18 at initial intake. 

The inclusion criteria for the control group required patients to (1) have a confirmed diagnosis of ADHD made by a psychiatrist according to the DSM-5 criteria; (2) have no past or present history of self-harm or suicide attempts as self-reported on the Dr. Sadek Psychiatric Assessment Intake Form; and (3) be males and females over the age of 18 at initial intake. 

**Figure 1:**
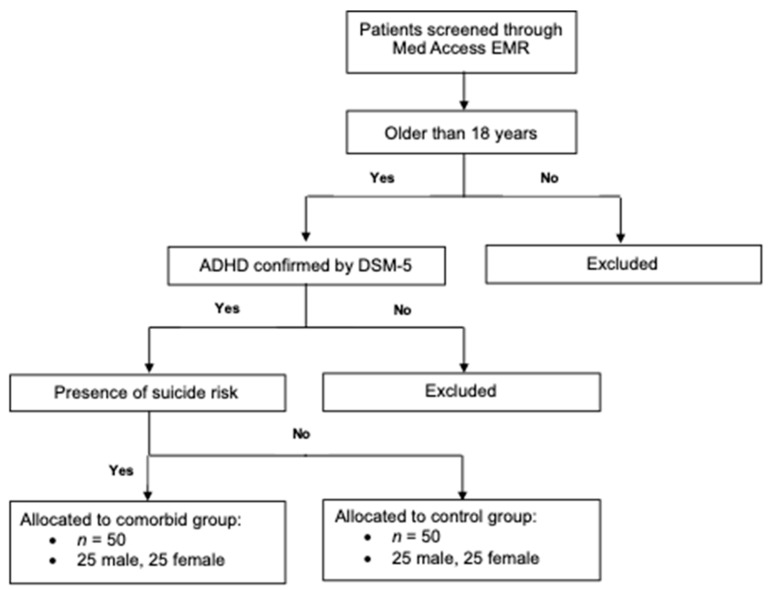
Inclusion criteria determined the control and experimental study groups. Patient electronic medical records (EMRs) were screened using the Med Access EMR software (version 5.14) starting on 1 January 2023. Patients were excluded from the study if they met any of the outlined exclusion criteria. Both sets of inclusion criteria were applied to the remaining population of adults with a confirmed diagnosis of attention deficit/hyperactivity disorder (ADHD) to determine the control (no suicide risk) and experimental groups (suicide risk), each with 50 patients.

The exclusion criteria for the study included (1) male and female patients who were under the age of 18 during initial intake at the psychiatric clinic and (2) patients who did not provide consent for their de-identified medical data to be used in scientific research. Figure 1 outlines how the inclusion and exclusion criteria were applied to the overall study population. By applying the exclusion criteria first, all the ineligible patients were removed, leaving a cohort of adults with a confirmed diagnosis of ADHD. Suicide risk was then assessed before allocating patients to either the control or experimental group.

The first 50 patients who met the inclusion criteria for each group (ADHD patients with suicide risk vs. ADHD patients without suicide risk) at the outpatient psychiatric clinic were included in this study, resulting in a sample size of 100. The patient records were sequentially screened from 1 January 2023 to the point at which both study groups comprised 50 participants or to 31 August 2023 (the end of the data collection period). The inclusion of adult ADHD patients with and without suicide risk was necessary to obtain the medical data needed to answer our study objectives. Moreover, given that several studies investigating the relationship between ADHD and suicide risk have been completed on younger patient populations [21,27], we decided that the patients should be 18 years or older. Our study also investigates the importance of psychiatric comorbidities, such as borderline personality disorder (BPD), binge eating disorder (BED), generalized anxiety disorder (GAD), and depression in the development of suicidal behaviours. BED is typically challenging to diagnose in children and adolescent populations because caloric intakes constantly change during development, and it may be difficult to define what a large amount of food is [28].

### 2.4. Data Collection

The patients’ medical data and PHI were collected through the outpatient psychiatric clinic’s Med Access EMR account. Both the primary and supervising investigator had authorized access to the charting system and the patients’ electronic medical records. Our access to their medical records was permitted, as the waiver of consent and privacy intake form was approved by the Nova Scotia REB. Therefore, no additional consent forms were required for us to use their PHI in this study. 

The data that were collected included (1) demographic information; (2) self-reported information on psychosocial factors, symptoms associated with psychiatric disorders, and developmental risk factors from the Dr. Sadek Psychiatric Assessment Intake Form; and (3) psychiatrist-reported information regarding psychiatric disorder diagnoses and additional risk factors. The Dr. Sadek Psychiatric Assessment Intake Form is based on the criteria outlined in the DSM-5 and ensures that additional psychiatric disorders are correctly identified and diagnosed. 

Demographic information like age and sex was self-reported and collected. Eight comorbid disorders were investigated in addition to ADHD and included BPD, BED, GAD, schizophrenia, depression, obsessive–compulsive disorder (OCD), substance use disorder, and alcohol use disorder. Information on self-reported past and present medical problems; drug and alcohol history; family and psychiatric history; challenges with personal/social abilities; and the inability to maintain relationships and employment was collected. The total number of antidepressant medication trials was gathered by the psychiatrist. Adversity factors were also assessed and consisted of prenatal and perinatal risks, including smoking, maternal alcohol consumption, and birth complications. Lastly, adverse childhood experiences like sexual abuse, physical abuse, academic difficulties, bullying, parental separation, and a history of delinquent and reckless behaviours were compared. 

To uphold the ethical duty of confidentiality and respect our patients’ right to privacy, we implemented several measures to ensure that the patient data were collected and stored in the most de-identified form possible. After the patient electronic medical records were identified, their data were organized in a Microsoft Excel^®^ spreadsheet before any identifying information associated with the data was erased. Patients were then assigned an anonymous study participant number that was completely unlinked to their medical data. The Microsoft Excel^®^ spreadsheet was protected with a highly secure password and was stored in a locked office at the supervising investigator’s clinic. Only the supervising investigator had access to the password-protected desktop computer, and both the primary and supervising investigator had access to the password-protected patient data spreadsheet. 

### 2.5. Statistics

In this study, we collected quantitative data that were electronically organized in a password-protected Microsoft Excel^®^ spreadsheet. Using version 28.0.1.1 of the IBM Statistical Package for the Social Sciences (SPSS) software, our non-parametric quantitative data were analyzed. The primary research question was answered using both the Chi-Square test of independence and the Mann–Whitney U Test. The Chi-Square test was used to identify significant relationships between two nominal variables, while the Mann–Whitney U Test was used to determine whether there were significant differences between two groups in terms of either their ordinal or continuous data. All the secondary research questions were answered using the same statistical tests. 

## 3. Results

The age range of the overall sample was 18 to 58, with a mean age of 31.37. The control (19–47) and experimental (18–58) groups had different age ranges but a similar mean age. The study sample consisted of equal numbers of males and females, with 25 male and 25 female participants in each study group. The control group had a confirmed primary diagnosis of ADHD without suicide risk. Conversely, the experimental group had a confirmed primary diagnosis of ADHD with suicide risk. In the control group, the inattentive ADHD presentation was the most common, with 54% of the participants diagnosed with it compared to those with the combined ADHD presentation, which occurred in only 46% of the participants. In the experimental group, the combined ADHD presentation was the most common, with 56% of the participants diagnosed this way, while the inattentive and hyperactive–impulsive presentations were less common (40% and 4%, respectively). Moreover, the study recorded eight comorbid psychiatric disorders that were noted in the participants. The most common psychiatric comorbidity in the study participants was GAD, occurring in about 97% of the study population, with both groups being relatively similar. Next was BPD, which was diagnosed in 52% of the study participants, with 18% of the participants in the control group being diagnosed compared to 86% in the experimental group. BED was diagnosed in 12% of the study sample; however, all these diagnoses were made in the experimental group and not the control group. All the other comorbid psychiatric disorders were similar among the control and experimental study groups. For the participants in the experimental group, the most common suicidal risk factor was active suicidal ideation, which was reported by 76% of the participants. The next most commonly reported factor was a recent history of self-harm (66%), followed by having a current suicidal plan (20%). Table 1 describes the characteristics of the study sample.

**Figure 2:**
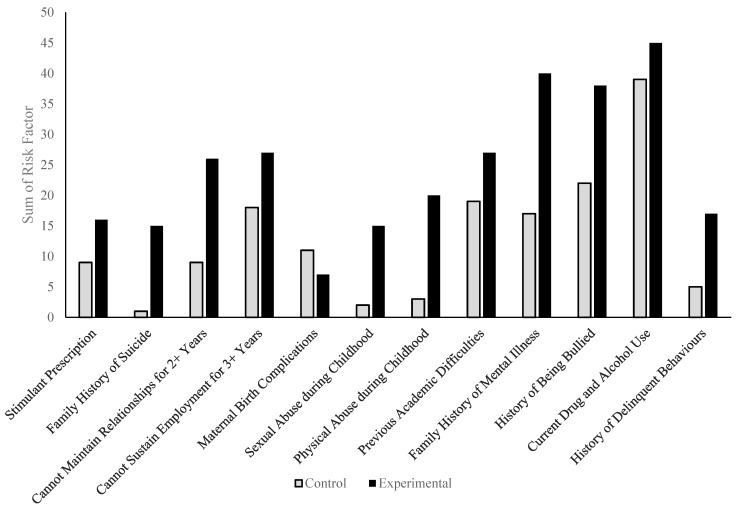
Patients in the comorbid group were more likely to report each risk factor, besides maternal birth complications. Each patient-reported risk factor was reported as either a 1 (present) or 0 (absent). The total sum for each risk factor in either the control or comorbid study groups was calculated to determine the overall sum of risk factors for each category.

Within the study sample, the most commonly reported physician and patient risk factors were current drug and alcohol use (85%), a history of being bullied (60%), and a family history of mental illness (57%). Each risk factor was more likely to be reported in the experimental group (90%, 76%, and 80%, respectively) compared to the control group (78%, 44%, and 34%, respectively). The majority of the risk factors were more common in the experimental group compared to the control group; however, maternal birth complications were higher in the control group (22%) than in the experimental group (14%). The parental separation rates were similar for both groups, as 50% of the control group reported them, while they were reported for 56% of the experimental group. In the control group, 38% had academic difficulties, 36% were unable to sustain employment for more than 3 years, 18% could not maintain relationships for more than 2 years, 18% were on a stimulant prescription, 6% had experienced physical abuse during childhood, 10% had a history of reckless or delinquent behaviour, 4% had experienced sexual abuse during childhood, and 2% reported a family history of suicide. In the experimental group, 54% had academic difficulties, 54% were unable to sustain employment for more than 3 years, 32% were on a stimulant prescription, 40% had experienced physical abuse during childhood, 34% had a history of reckless or delinquent behaviour, 30% had experienced sexual abuse during childhood, and 30% reported a family history of suicide. Prenatal maternal smoking and alcohol consumption in both groups were close to 0%. Table 2 shows the overall number of physician- and patient-reported risk factors in the study sample, while Figure 2 depicts the sum of the patient-reported risk factors.

To examine whether there were significant differences between the specific risk factors in the control and experimental groups, Chi-Square testing was used. For each risk factor, contingency tables were made; the participants in each study group were characterized as either having (1) or not having (0) any specific risk factor, resulting in the collection of nominal data. Using a significance level of 0.05 (α = 0.05), seven of the fourteen risk factors assessed revealed statistical significance. The following risk factors were significantly associated with a principal diagnosis of ADHD and suicide risk: family history of mental illness (*p* = 0.000003), family history of suicide (*p* = 0.000134), inability to maintain relationships for 2+ years (*p* = 0.000365), sexual abuse during childhood (*p* = 0.000539), physical abuse during childhood (*p* = 0.000054), history of bullying (*p* = 0.0010908), and history of delinquent behaviours (*p* = 0.0037696). Conversely, a stimulant prescription (*p* = 0.518424), current drug and alcohol use (*p* = 0.101707), inability to sustain employment for 3+ years (*p* = 0.070440), prenatal drug or alcohol consumption (*p* = 1), maternal birth complications (*p* = 0.169384), previous academic difficulties (0.108462), and parental separation (*p* = 0.547785) were not significantly associated with a principal diagnosis of ADHD and suicide risk. Table 3 contains the *p*-values of the specific risk factors comparing the experimental group (ADHD with suicide risk) to the control group (ADHD without suicide risk). 

The study determined the overall number of risk factors for each participant by adding together each factor outlined in Table 3. These values were then compared between the control and experimental groups using the Mann–Whitney U test. The control group (ADHD without suicide risk) had a mean of 4.56 and a mean rank of 38.65, while the experimental group (ADHD with suicide risk) had a mean of 6.32 and a mean rank of 62.95. The test produced a *p*-value of 0.000013, indicating a significant difference between the overall number of risk factors for the ADHD patients with suicide risk (experimental) and the ADHD patients without suicide risk (control). Table 4 outlines the differences in the sum of risk factors, the mean number of risk factors, and the mean rank between the control and experimental groups, providing a *p*-value (α = 0.05) to indicate whether the difference is significant. 

**Figure 3:**
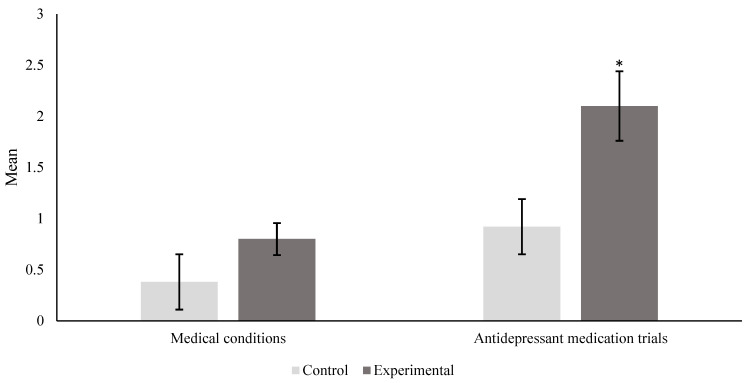
A greater number of antidepressant medication trials was significantly associated with suicide risk, whereas the number of medical conditions was not. The Mann–Whitney U test was used at a significance level of 0.05 to examine differences between the number of antidepressant medication trials and medical conditions. The figure contains standard error bars of the mean where * = *p* < 0.05.

The number of both medical conditions and antidepressant medication trials were examined using the Mann–Whitney U test with a significance level of 0.05 (α = 0.05). For each participant, their total number of medical conditions and antidepressant medication trials were summed and tabulated, resulting in the collection of scale data that could be ranked. For the number of medical problems, the mean rank for the control group was 45.83, while it was 55.17 for the experimental group, resulting in a *p*-value of 0.060585; this indicated that there was no significant association for medical conditions between the control and experimental groups. Conversely, for the number of antidepressant medication trials, the mean rank for the control was 44.28 compared to 56.72 for the experimental group, producing a *p*-value of 0.024972. This value is lower than the significance level (α = 0.05), indicating a significant association with antidepressant medication trials for both study groups. Table 5 contains the mean rank and *p*-value for both the number of medical conditions and antidepressant medication trials. Figure 3 includes the means with standard error (SE) bars for the control and experimental groups for both factors, indicating which differences are significant. 

Chi-square testing was used to determine whether there were significant differences in terms of specific psychiatric comorbidities between the control and experimental groups. The participants in either group either had (1) confirmed psychiatric diagnosis/diagnoses or did not (0) have any psychiatric comorbidities. All the psychiatric disorders were assessed at a significance level of 0.05. Two of eight psychiatric comorbidities showed a significant difference between the ADHD patients with suicide risk and those without suicide risk, which included BPD (*p* = 1.0074 × 10^−11^) and BED (*p* = 0.011522). The following psychiatric disorders did not show any significant differences: generalized anxiety disorder (*p* = 0.557734), schizophrenia (*p* = 1.000000), depression (*p* = 0.168669), obsessive–compulsive disorder (*p* = 1.000000), substance use disorder (*p* = 0.399703), or alcohol use disorder (*p* = 0.646355). Table 6 shows the *p*-values obtained using the Mann–Whitney U test for each psychiatric comorbid disorder as compared to the control group. 

The overall number of comorbid psychiatric disorders was also compared. For each participant, their number of psychiatric disorders was added and then compared between groups using the Mann–Whitney U-test with a significance level of 0.05. The control group’s mean for psychiatric disorders was 1.34 with a mean rank of 35.46. Conversely, the experimental group’s mean was 2.22, having a mean rank of 65.54. The results of the test revealed a *p*-value 3.1164 × 10^−8^, which indicates a significant relationship. Table 7 outlines the sum of the overall psychiatric comorbidities, the mean number of psychiatric disorders, and the mean rank including the *p*-value determined using the Mann–Whitney U test.

## 4. Discussion 

The purpose of this retrospective study was to examine a population of adults with a confirmed diagnosis of ADHD and determine whether there were significant differences between those with suicide risk and those without suicide risk. This retrospective study gathered data from the Dr. Sadek Psychiatric Assessment Intake Form in a natural clinical setting. We hypothesized that adult ADHD patients with suicide risk (experimental) would have a greater number of psychiatric comorbidities, medical problems, and antidepressant medication trials and poorer social abilities compared to adult ADHD patients without suicide risk (control). Our main findings were that the ADHD patients with suicide risk were (1) significantly associated with seven patient-reported risk factors: family history of mental illness, family history of suicide, inability to maintain relationships for 2 or more years, sexual abuse during childhood, physical abuse during childhood, history of being bullied, and a history of delinquent behaviours; (2) significantly more likely to have comorbid diagnoses of BPD or BED; (3) significantly associated with a higher number of antidepressant medication trials; and (4) significantly associated with a greater mean number of risk factors and psychiatric comorbidities. However, there was no significant difference in the number of medical conditions between the control and experimental groups. Therefore, our hypothesis is mostly supported by our findings described above, in addition to the data outlined in Table 3, Table 4, Table 5, Table 6 and Table 7. 

Generally, the breakdown of the ADHD presentations in our study sample is consistent with other studies examining populations composed of adults with ADHD; however, the combined presentation is often more prevalent in these studies, with there being a larger difference between those who have the combined presentation and the inattentive presentation [7,29]. Considering that impulsivity has previously been referenced in the literature as a reason for the development of suicidal ideation and behaviours [9,10], we expected to see higher rates of the hyperactive–impulsive presentation, which was not the case in our findings. This discrepancy could be explained by age-dependent differences in ADHD presentations, as children diagnosed with ADHD are more likely to present with hyperactive and impulsive symptoms compared to adults; however, the age-dependent remission rates for hyperactive and impulsive symptoms are much greater, while inattentive symptoms are less likely to remit and often persist into adulthood [5]. This shift in presentation reflects the changes that were made in the DSM-5 from subtypes to presentations to reflect the dynamic and fluid nature of ADHD diagnoses [8]. This difference could also potentially be explained by the origin of our study sample, as it involved patients from a specific geographical location who had visited an outpatient psychiatric clinic in Dartmouth, Nova Scotia. 

Based on our current understanding, this is one of the few studies examining several risk factors associated with a population of ADHD patients with suicide risk, using a sample of ADHD-confirmed patients as the control group. We report several interesting findings in terms of the risk factors, many being consistent with the current literature. Two genetic factors that were associated with suicide risk were a family history of both mental illness and suicide (Table 3). Previous research has described the importance of familial risks, with differing levels of relatedness. Essential to this are shared genetics that are passed onto various relatives, as the greatest risk has been identified for first-degree relatives because genetic factors are significantly more likely to be shared [24]. In fact, there were statistically significant differences in the risk between first-degree and second-degree relatives, while maternal and paternal half-siblings showed no significant differences, providing little support for shared environmental factors [24]. Important to note is that genetic factors not only play a role in the development of suicidal behaviour and risk but also ADHD itself [24], which is likely related to impulsivity. A potential explanation for this increased genetic risk involves individual environmental factors, which likely give rise to many genetic variants in the genes associated with impulsivity. Additionally, there is no one “impulsivity gene”, and rather clusters of pleiotropic genes are involved in influencing impulsive behaviour [30], in addition to environmental influences. 

Another significant finding that was associated with suicide risk was the inability to maintain relationships for 2 years or more. This finding is consistent with other studies examining precipitating factors for suicide risk in patients with ADHD. One study describes how social isolation plays an important role in suicidal risk in both children and adolescents [18]. The effects could likely continue into adulthood, where an inability to maintain relationships induces feelings of social isolation, thus increasing the risk of suicidal behaviours. Moreover, after consecutive unsuccessful attempts to maintain relationships, one may develop feelings of hopelessness, which is another significant risk factor that is associated with suicide intent and risk [31]. 

Additionally, we found that both childhood physical and sexual abuse were significantly associated with suicide risk. Consistent with the current literature, children exposed to these abusive behaviours are at a significantly higher risk of suicide [18,20,32]; however, this finding extends throughout several age groups, having impacts on adults (20–64 years old) and members of the geriatric population (65+ years old) [18,33]. A history of abuse, both sexual and physical, has been described as being a greater predictor of suicide risk and behaviour when compared to other risk factors [33]. One possible explanation for this increased risk involves the subsequent impact that abuse has on multiple factors, increasing the risk of BPD, impulsive aggression, and interpersonal sensitivity [34]. With the long-standing negative effects of abuse and the increased chance of engaging in maladaptive behaviours, ensuring that people from all age groups remain protected and safe is essential to reduce suicide risk. More research is needed on this relationship in a population of adults with a confirmed diagnosis of ADHD. 

This study further demonstrates a possible significant association between suicide risk in ADHD patients and a history of delinquent behaviours, which are considered behaviours that contrast with societal norms. One scientific article describes how despite comorbid disorders having a large role in the development of suicidal behaviour, both delinquency and substance misuse are very important factors [35]. Moreover, behaviours that are consistent with conduct disorders also have a significant impact on suicide risk, as they have been previously linked with suicidal behaviours and intent [33]. Conduct disorders can further exacerbate deviancy and drug abuse, both of which are factors that have been previously linked to suicide [36]. In addition, if these delinquent behaviours lead to traumatic childhood experiences, we see subsequent changes in risk, as these experiences could be internalized and could potentially lead to mental health disorders. We suggest that researchers examine the association between childhood delinquent behaviours and suicide risk in ADHD patients to improve their clinical profiles. 

Uniquely, this study examined the overall number of antidepressant medication trials for each participant. To our knowledge, this is the first study to describe the negative effects of an increasing number of antidepressant medication trials. Suicide risk in ADHD patients was elevated in those who more frequently changed their antidepressant medications due to the unsuccessful resolution of their current symptoms. This finding is interesting, as it reveals that trialing various antidepressant medications can have counterintuitive effects, even if the long-term goal is to find an effective treatment. This is also important when considering drug regimens for patients with treatment-resistant depression, a form of depression that does not respond to various first-line therapeutic options [37]. Rather than continuously trying alternative antidepressant medications, caution should be taken when prescribing these medications to patients with and without ADHD to prevent the chance of significantly increasing suicide risk. Conversely, for patients who effectively respond to antidepressant medications, there is a lot of controversy on the effects of antidepressants on suicide risk, with some literature suggesting that antidepressants increase risk [38]. We advise that further research examines whether this relationship persists in populations of adults without ADHD using larger sample sizes to ensure that statistically significant results are found. 

Our findings were generally consistent with other studies describing the overall involvement of comorbid psychiatric disorders in the development of suicidal ideation and risk [16,20,23]. The literature often suggests that an important psychiatric disorder that largely influences the risk of suicide is major depressive disorder (MDD), a finding that was not reflected in our study with our sample size. Several participants in the experimental group often reported feeling depressed or having low mood but did not meet the criteria for MDD as outlined in the DSM-5. A link between ADHD and suicide attempts has been established in the literature, with many authors describing how the detrimental effects of MDD on mood and behaviour are driving factors in this relationship [16,39]. Conversely, in similar populations of ADHD patients, the role of MDD in the development of suicidal ideation has been less supported [23]. The lack of MDD diagnoses in our study could potentially be explained by the group differences and the time of data collection, as the majority of our data collection occurred in January 2023, when people were slowly shifting into more regular lives after the lifting of COVID-19 restrictions. 

Interestingly, BPD and BED were significantly more likely to be reported by the participants in the experimental group who had a diagnosis of ADHD and suicide risk compared to the control group. Despite not being a primary factor associated with the development of suicide risk, these findings are consistent with other studies. One study described several comorbid diagnoses being associated with at-risk ADHD patients who had previously attempted suicide [40]. In this study, BPD was listed as the third most common comorbid psychiatric diagnosis after MDD and substance abuse disorder. BPD was significantly more prevalent in patients who were at risk of suicide due to at least one previous suicide attempt [40]. An important feature of BPD is impulsivity, which likely explains why people with this disorder are more likely to act on any intrusive suicidal ideations or plans. A study examining impulsivity in patients with BPD revealed that people with a confirmed BPD diagnosis were more likely to attempt suicide multiple times, partake in frequent suicidal thoughts, and engage in repetitive patterns of self-cutting [41]. Therefore, this could help explain why there was such a significant difference in the diagnosis of BPD between the control and experimental groups, with those in the experimental group being significantly more likely to have BPD. 

Furthermore, BED was also significantly associated with ADHD patients who presented with suicide risk. BED is a behavioural disorder that involves consuming an objectively large amount of food and is characterized by a sense of lost control and an inability to manage impulses [28]. Important to note is that similar to BPD, BED also has an impulsive component, which would explain why it is also associated with suicide risk in ADHD patients. This finding is reflected in a study describing which comorbid disorders were significantly associated with suicidal behaviour and risk in ADHD patients [40]. Moreover, several environmental factors influence the development of BED and include things like severe adverse childhood experiences, disturbances in family functioning, and weight-related teasing and bullying [42,43]. Some of these risk factors were also significantly associated with suicide risk in the experimental group, such as a history of bullying. Thus, several precipitating factors influence BED, supporting the significant association between bullying in childhood and suicide risk in the experimental group. 

None of the other highlighted psychiatric disorders were significantly different between the control and experimental groups. Both alcohol and substance use disorders were barely identified in our study sample; however, many participants did report frequent alcohol and substance use. This lack of DSM-5 diagnoses is likely explained by a lack of risky use and impaired control while consuming, as many of the participants seemed to only casually use both alcohol and substances. On the other hand, a very common diagnosis in both the control and experimental groups was GAD. The frequency of GAD could be explained by the fact that the patients enrolled in this study visited the outpatient psychiatric clinic to determine what disorders were causing them such difficulties and distress in their day-to-day functioning. With anxiety disorder being one of the world’s most common psychiatric disorders [44], this could help explain why the majority of our patient population was diagnosed with it. 

Several other risk factors that we identified were not significantly associated with suicide risk in the adult ADHD patients. Furthermore, we found no significant association between the number of medical conditions and suicide risk in ADHD patients. The lack of significant findings between these factors and suicide suggests that they may not be associated with suicide risk in ADHD patients or may just have very small influences that act synergistically with other factors. Additional research should examine these factors in a similar patient sample to confirm our findings. 

Regarding all these risk factors and psychiatric comorbidities, it is important to consider how they all interact with one another. None of these risk factors likely exist in isolation but rather involve the interplay of biological, social, and psychological factors. Biological risk factors were not examined in this study; however, these may genetically predispose certain patients to particular risk factors, subsequently increasing their risk of ADHD and/or suicide [24]. Further, we examined a variety of both social and psychological risk factors within this study but were unable to examine how they interfered or interacted with each other. We suggest that future studies attempt to determine the complex interplay of these factors to determine whether significant associations still exist. 

### 4.1. Limitations and Future Directions

As this was a pilot study, the validity of our findings is limited by our small sample size (*N* = 100). Therefore, the several statistically significant associations we found between suicide risk in ADHD patients and (1) overall risk factors, (2) antidepressant medication trials, and (3) psychiatric comorbidities cannot be described or interpreted as definitive correlations. Despite this limitation, our study reveals several interesting findings that provide support to other studies and even suggest novel findings on an association between the overall number of antidepressant medication trials and suicide risk in ADHD patients. The *p*-values were not adjusted for multiple comparisons, increasing the likelihood of type I errors. Considering that clinician judgment was required for certain aspects of the data collection, this also poses a minor limitation when considering the involvement of psychiatric disorders. Another limitation surrounds the collection of memories established in childhood, as this allows for poor recall of past experiences to play a role in the accuracy of some of the reported risk factors. This may be addressed by changing the study design from a retrospective chart study to a retrospective longitudinal study, where data are collected from existing medical records. Additionally, this retrospective nature further limits our ability to determine causation and ask further questions for clarification regarding certain factors or conditions. Therefore, we were only able to determine whether significant associations existed in our study. 

The use of convenience sampling reduced our ability to generalize the data, as all the patients included in this study came from a single clinic. Moreover, their generalizability was further limited as the patients came from similar geographic backgrounds. Future research should focus on examining these risk factors in addition to others using a larger more representative sample of ADHD patients to improve the generalizability and validity of the data. For example, rather than using convenience sampling, data could be collected from multiple clinics across the province using patients with diverse backgrounds to enhance the ability to make generalizations about the data. Introducing the collection of protective factors would also present interesting findings, helping to improve the clinical profiles of ADHD patients. Moreover, examining this relationship using a control group of ADHD patients without suicide risk is important, as ADHD is a very common neurodevelopmental disorder, and having a better understanding of its complexities is essential in trying to effectively deal with its symptoms. Ultimately, treatment methods need to be specialized to this study sample to ensure effective management of both ADHD symptoms and suicidal factors, indicating a need for additional research in this area. 

### 4.2. Conclusions

This study highlighted the impact of various risk factors in patients with ADHD and suicide risk, using a control group of adults with ADHD and no suicide risk. Our results showed significant associations between (1) two psychiatric comorbidities, (2) seven patient-reported risk factors, and suicide risk in ADHD patients. Interestingly, we found significant associations linking an increased number of antidepressant medication trials to suicide risk, suggesting that changes should be considered when prescribing this class of medications. Several other risk factors did not show statistical significance, suggesting either their absent or minimal effect on the development of suicide risk in this patient sample. An understanding of these factors will help develop the clinical profiles of these patients, allowing for more effective identification of suicide risk so that treatment plans can be established. Given the overall lack of research examining these risk factors in a similar patient population, more research should be focused on this area. Furthermore, stratifying for gender would present interesting findings in terms of risk factors and psychiatric comorbidities and thus should be examined in future studies.

## Figures and Tables

**Table 1:** Diagnostic and sociodemographic characteristics of the study sample (*N* = 100). Diagnostic and sociodemographic data were collected from Med Access electronic medical records (EMRs) and the Dr. Sadek Psychiatric Assessment Intake Form and tabulated to better characterize the study sample.

Characteristics	Study (*N* = 100)	Control (*N* = 50)	Experimental (*N* = 50)
**Age**			
Range	18–58	19–47	18–58
Mean	31.37	31.28	31.46
SD	8.45	8.25	8.74
**Sex**			
Male	50	25	25
Female	50	25	25
**ADHD Diagnosis**			
Combined	51	23	28
Inattentive	47	27	20
Hyperactive–Impulsive	2	0	2
**Suicide Risk Factors**			
Current Suicidal Ideation (SI)	38	0	38
Suicidal/Harmful Plan	10	0	10
Previous Attempts to Harm Self	33	0	33
**Comorbid Disorders**			
Borderline Personality Disorder (BPD)	52	9	43
Generalized Anxiety Disorder (GAD)	97	48	49
Schizophrenia	1	1	0
Depression	5	1	4
Obsessive–Compulsive Disorder (OCD)	6	3	3
Binge Eating Disorder (BED)	6	0	6
Substance Use Disorder (SUD)	6	2	4
Alcohol Use Disorder (AUD)	5	3	2

**Table 2:** Patient-reported risk factors for the study sample and control and comorbid groups. Specific psychosocial risk factors were collected from the Dr. Sadek Psychiatric Assessment Intake Form, which contains self-reported patient information from the first initial intake. The presence of a risk factor was denoted as a 1, whereas the absence was denoted as a 0. After data tabulation, the overall count for each risk factor was divided into its respective category for statistical analysis.

Risk Factor	Study (*N* = 100)	Control (*N* = 50)	Experimental (*N* = 50)
Stimulant Prescription	25	9	16
Current Drug and Alcohol Use	84	39	45
Family History of Mental Illness	57	17	40
Family History of Suicide	16	1	15
Cannot Maintain Relationships for 2+ Years	35	9	26
Cannot Sustain Employment for 3+ Years	45	18	27
Prenatal Drug/Alcohol Consumption	2	1	1
Maternal Birth Complications	18	11	7
Sexual Abuse During Childhood	17	2	15
Physical Abuse During Childhood	23	3	20
Academic Difficulties	46	19	27
History of Being Bullied	60	22	38
Parental Separation	53	25	28
History of Delinquent Behaviours	22	5	17

**Table 3:** Seven of fourteen risk factors were significantly associated with suicide risk in adult ADHD patients. The total number of risk factors for each category was calculated for both the study and comorbid groups, each containing 50 patients. The presence of a risk factor was denoted as 1 whereas the absence was noted as a 0. The Chi-Square test for association was used to analyze the differences between the two independent samples’ nominal data. The *p*-values were calculated using a significant level of 0.05 (95% CI), where any bolded numbers represent statistical significance (*p* < 0.05).

Risk Factor	Control (*N* = 50)	Experimental (*N* = 50)	*p*-Value
Stimulant Prescription	9	16	0.518424
Current Drug and Alcohol Use	39	45	0.101707
Family History of Mental Illness	17	40	**0.000003**
Family History of Suicide	1	15	**0.000134**
Cannot Maintain Relationships for 2+ Years	9	26	**0.000365**
Cannot Sustain Employment for 3+ Years	18	27	0.070440
Prenatal Drug/Alcohol Consumption	1	1	1.000000
Maternal Birth Complications	11	7	0.169381
Sexual Abuse During Childhood	2	15	**0.000539**
Physical Abuse During Childhood	3	20	**0.000054**
Academic Difficulties	19	27	0.108462
History of Being Bullied	22	38	**0.001091**
Parental Separation	25	28	0.547785
History of Delinquent Behaviours	5	17	**0.003770**

**Table 4:** The comorbid study group has significantly greater numbers of risk factors compared to the control group. The total number of risk factors for each group was calculated by combining the overall count of each independent risk factor. The ordinal data were assessed between the two independent groups using the Mann–Whitney U test at a significance level of 0.05 (95% CI), where a bolded number indicates statistical significance. This statistical test uses the mean rank rather than the mean to account for any outliers.

Group	Total Sum of Risk Factors	Mean	Mean Rank	*p*-Value
Control (*N* = 50)	228	4.56	38.05	
Experimental (*N* = 50)	316	6.32	62.95	**0.000013**

**Table 5:** A greater number of antidepressant medication trials is associated with suicide risk and ADHD in adult patients. The Mann–Whitney U test was used to compare the number of antidepressant medication trials and medical conditions between the control and comorbid groups. The analysis was conducted at a significance level of 0.05 (95% CI), where bolded numbers represent statistical significance (*p* < 0.05).

Factor	Control (*N* = 50) Mean Rank	Experimental (*N* = 50) Mean Rank	*p*-Value
Number of Medical Conditions	45.83	55.17	0.060585
Number of Antidepressant Medication Trials	44.28	56.72	**0.024972**

**Table 6:** Borderline personality disorder (BPD) and binge eating disorder (BED) were significantly associated with suicide risk in adult ADHD patients. The presence of a comorbid psychiatric disorder was noted as a 1, whereas its absence was marked as a 0. The Chi-Square test of association was used at a significance level of 0.05 to determine whether there were significant differences between the two independent study groups. Bolded numbers represent statistical significance (*p* < 0.05).

Comorbid Disorder	Control (*N* = 50)	Experimental (*N* = 50)	*p*-Value
Borderline Personality Disorder (BPD)	9	43	**1.0074 × 10^−11^**
Generalized Anxiety Disorder (GAD)	48	49	0.557734
Schizophrenia	1	0	1.000000
Depression	1	4	0.168669
Obsessive–Compulsive Disorder (OCD)	3	3	1.000000
Binge Eating Disorder (BED)	0	6	**0.011522**
Substance Use Disorder (SUD)	2	4	0.399703
Alcohol Use Disorder (AUD)	3	2	0.646355

**Table 7:** The comorbid group has significantly greater numbers of psychiatric disorders as compared to the control group. The total number of psychiatric disorders was calculated by adding up the total for each patient in the control and comorbid study groups. The Mann–Whitney U test was used at a significance level of 0.05 to determine whether significant differences existed between the two independent study groups. Bolded numbers represent statistical significance (*p* < 0.05).

Group	Sum of Psychiatric Comorbidities	Mean	Mean Rank	*p*-Value
Control (*N* = 50)	67	1.34	35.46	
Experimental (*N* = 50)	111	2.22	65.54	**3.1164 × 10^−8^**

## Data Availability

The data that were presented in this study are available upon request from the authors. The data are not publicly available due to confidentiality, as they may contain personal health information on the study participants.
